# Ketone bodies: A double‐edged sword for mammalian life span

**DOI:** 10.1111/acel.13833

**Published:** 2023-04-14

**Authors:** Issei Tomita, Hiroaki Tsuruta, Mako Yasuda‐Yamahara, Kosuke Yamahara, Shogo Kuwagata, Yuki Tanaka‐Sasaki, Masami Chin‐Kanasaki, Yukihiro Fujita, Eiichiro Nishi, Hideki Katagiri, Hiroshi Maegawa, Shinji Kume

**Affiliations:** ^1^ Department of Medicine Shiga University of Medical Science Tsukinowa‐cho, Seta Otsu Shiga 520‐2192 Japan; ^2^ Department of Pharmacology Shiga University of Medical Science, Tsukinowa‐cho, Seta Tsukinowa‐cho, Seta Otsu Shiga 520‐2192 Japan; ^3^ Department of Metabolism and Diabetes Tohoku University Graduate School of Medicine 2‐1 Seiryo‐machi, Aoba‐ku Sendai Miyagi 980‐8574 Japan

**Keywords:** Hmgcs2, ketone body, longevity, low‐carbohydrate ketogenic diet

## Abstract

Accumulating evidence suggests health benefits of ketone bodies, and especially for longevity. However, the precise role of endogenous ketogenesis in mammalian life span, and the safety and efficacy of the long‐term exogenous supplementation of ketone bodies remain unclear. In the present study, we show that a deficiency in endogenous ketogenesis, induced by whole‐body *Hmgcs2* deletion, shortens life span in mice, and that this is prevented by daily ketone body supplementation using a diet containing 1,3‐butanediol, a precursor of β‐hydroxybutyrate. Furthermore, feeding the 1,3‐butanediol‐containing diet from early in life increases midlife mortality in normal mice, but in aged mice it extends life span and prevents the high mortality associated with atherosclerosis in *ApoE*‐deficient mice. By contrast, an ad libitum low‐carbohydrate ketogenic diet markedly increases mortality. In conclusion, endogenous ketogenesis affects mammalian survival, and ketone body supplementation may represent a double‐edged sword with respect to survival, depending on the method of administration and health status.

## Graphical abstract

The present study demonstrates that endogenous ketogenesis is essential for long‐term mammalian survival, which was revealed by a study using *Hmgcs2* ^−/−^ mice, and that dietary administration of 1,3‐butanediol (1,3‐BD), a precursor of β‐OHB, increases midlife mortality in normal mice, but extends life span in aged mice or atherosclerotic mice. By contrast, ad libitum low‐carbohydrate ketogenic diet increases mortality in any mouse models. Thus, ketone bodies represent a double‐edged sword for mammalian survival.

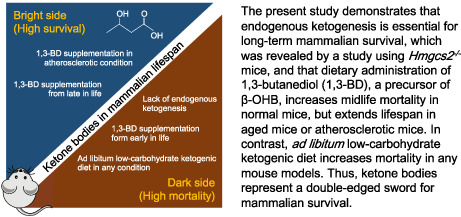

## 1 INTRODUCTION

Ketone bodies (KBs) represent an important glucose‐sparing energy source during fasting in mammals (Cahill et al., 1970; Reichard et al., 1974), and they have been suggested to be involved in the prolongation of life span induced by calorie restriction and low‐carbohydrate ketogenic diet (LCKD)‐feeding (Newman et al., 2017; Roberts et al., 2017; Stekovic et al., 2019). However, their precise role in mammalian longevity has long been debated because such dietary interventions cause many metabolic changes in addition to ketogenesis.

**FIGURE 1:**
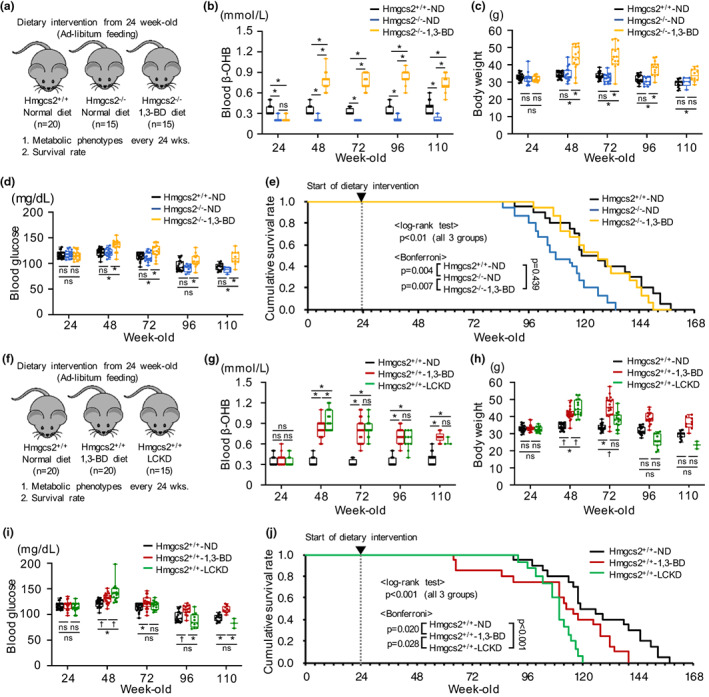
Effects of endogenous ketogenesis and dietary KB supplementation on mouse survival rate. (a) Study protocol for determining the life span of *Hmgcs2* ^*+/+*^ mice fed a normal diet (ND; *n* = 20) and *Hmgcs2* ^*−/−*^ mice fed either an ND (*n* = 15) or a 1,3‐butanediol (1,3‐BD)‐containing diet (*n* = 15). (b) β‐OHB concentration during the study period. (c) Change in body weight. (d) Blood glucose concentration. (e) Cumulative survival rate. (f) Study protocol for determining the life span of *Hmgcs2* ^*+/+*^ mice fed an ND (*n* = 20), a 1,3‐BD diet (*n* = 20), or a low‐carbohydrate ketogenic diet (LCKD), which contained 4.5% carbohydrate, 80.8% fat, and 14.7% protein (calorie %) (*n* = 15), from 24 weeks of age. (g) β‐OHB concentration. (h) Change in body weight. (i) Blood glucose concentration. (j) Cumulative survival rate. *p < 0.01, †*p* < 0.05; ns, not significant.

In the present study, the survival of *Hmgcs2* ^−/−^ mice that we generated previously (Tomita et al., 2020), which are incapable of endogenous ketogenesis, was assessed to evaluate the role of KBs in mammalian life span (Figure 1a). *Hmgcs2* deficiency increased the mortality rate in old age, and this was accompanied by lower blood beta‐hydroxybutyrate (β‐OHB) concentration, but no differences in body weight or blood glucose concentration (Figure 1b−e, Table S1). The reduction in life span of the *Hmgcs2* ^−/−^ mice was prevented by the dietary administration of 1,3‐butanediol (1,3‐BD), a chemical precursor of β‐OHB (Figure 1a,e). Higher mortality of *Hmgcs2* ^−/−^ mice during the weaning period has previously been reported^7^. Therefore, the present findings emphasize the importance of KBs for mammalian survival and imply that they have significant effects on life span during two time windows: just after birth and in old age.

The health benefits of KBs have recently been gaining attention (Cheng et al., 2019; Cotter et al., 2014; Cox et al., 2016; Fan et al., 2021; Mujica‐Parodi et al., 2020; Nielsen et al., 2019; Tomita et al., 2020; Torres et al., 2019). Therefore, there is an urgent need to determine whether long‐term KB supplementation is safe and extends life span. To answer this question, the effects on the life span of control *Hmgcs2* ^+/+^ mice of an LCKD, which contained 4.5% carbohydrate, 80.8% fat, and 14.7% protein (calorie %), and a 1,3‐BD‐containing diet from 24 weeks of age were examined (Figure 1f). LCKD‐fed mice exhibited higher mortality in old age, which was accompanied by rapid decreases in body weight and blood glucose concentration after midlife (Figure 1g−j, Table S2). The survival rate of the 1,3‐BD diet–fed mice was also lower, but there were differences in mortality from the LCKD‐fed mice at various time points (Figure 1j). The 1,3‐BD diet was associated with higher midlife mortality, but this difference disappeared when the normal diet (ND)‐fed mice began to die (between 90 and 110 weeks of age), such that their mortality in old age was similar to that of ND‐fed mice (Figure 1j).

**FIGURE 2:**
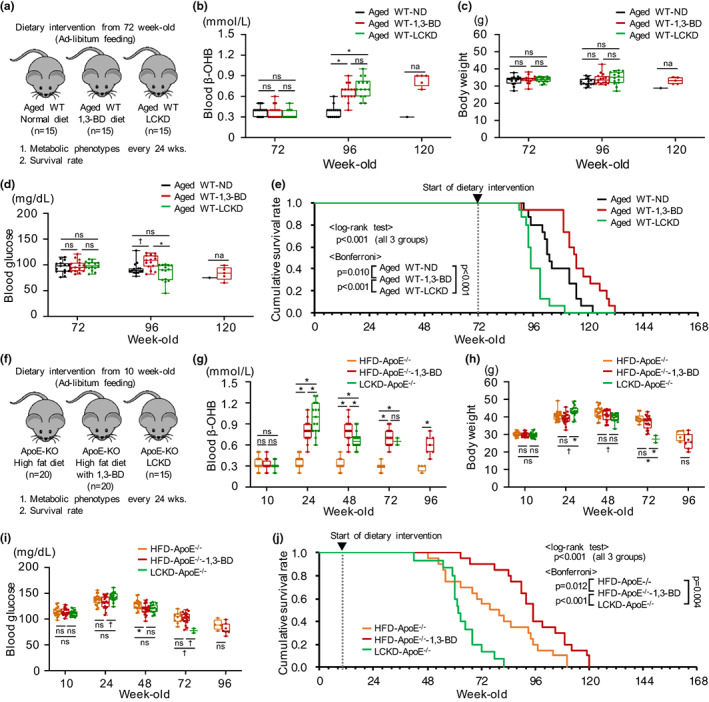
Effects of KB supplementation on the survival rate of aged mice and young *ApoE* ^*−/−*^ mice. (a) Study protocol for determining the life span of aged wild‐type (WT) C57BL/6J mice fed a normal diet (ND; *n* = 15), a 1,3‐butanediol (1,3‐BD)‐containing diet (*n* = 15), or a low‐carbohydrate ketogenic diet (LCKD), which contained 4.5% carbohydrate, 80.8% fat, and 14.7% protein (calorie %) (*n* = 15). (b) β‐OHB concentration during the study period. (c) Change in body weight. (d) Blood glucose concentration. (e) Cumulative survival rate. (f) Study protocol for determining the life span of *ApoE* ^*−/−*^ mice fed either an ND (*n* = 20), a 1,3‐BD diet, (*n* = 20) or an LCKD (*n* = 15). (g) β‐OHB concentration. (h) Change in body weight. (i) Blood glucose concentration. (j) Cumulative survival rate. *p < 0.01, ^†^ *p* < 0.05; ns, not significant; na, not available.

This finding led us to hypothesize that KB supplementation commencing just prior to old age might extend life span. To evaluate this possibility, the 1,3‐BD diet or LCKD was fed to C57BL/6J mice from 72 weeks of age (Figure 2a). Both interventions increased the blood β‐OHB concentration similarly, and had no marked effects on body weight or blood glucose levels (Figure 2b−d, Table S3). However, the 1,3‐BD diet extended the life span, whereas the LCKD shortened it (Figure 2e).

These findings led us to further hypothesize that the 1,3‐BD diet might extend life span, even in young mice, if they have organ damage. Therefore, we next determined the effects of KB administration on the life span of high‐fat diet (HFD)‐fed *ApoE* ^−/−^ mice with atherosclerosis‐related organ damage (Figure 2f). The 1,3‐BD diet and LCKD induced similar metabolic changes to those identified in the aged mice (Figure 2g−i, Table S4). The high mortality of the HFD‐fed *ApoE* ^−/−^ mice was reduced by the 1,3‐BD diet but worsened by the LCKD (Figure 2j).

The present results suggest that the timing and method of KB supplementation and health status have significant influences on the effects of KBs on mammalian life span. Given that *Hmgcs2* ^−/−^ mice had a lower survival rate in old age and that 1,3‐BD extended the life span of aged and *ApoE* ^−/−^ mice, KBs may have an important role in tissue repair, as suggested by recent studies (Cheng et al., 2019; Tomita et al., 2020). By contrast, 1,3‐BD diet from early life was associated with higher mortality, although the reason for this remains unclear. Thus, the potential for KBs to be used to promote human health (Chen et al., 2021) and their safety, particularly in younger individuals, requires further investigation. Also, further analysis regarding the cause of death is needed to elucidate the mechanisms by which KBs affect the life span.

The health benefits of LCKD remain the subject of debate. Time‐restricted or energy‐controlled LCKD feeding was reported to extend life span in mice (Newman et al., 2017; Roberts et al., 2017), whereas ad libitum LCKD feeding leading to much calorie intake shortened their life span in our study. Thus, the unrestricted LCKD seems to cancel out the health benefits of KBs and to be harmful. Because LCKDs are often used for weight management (Foster et al., 2003), the effects of long‐term LCKD consumption require careful monitoring.

In conclusion, it is possible that endogenous ketogenesis plays an important role in mammalian survival, although there may be sex differences in the effects. Furthermore, KB supplementation represents a double‐edged sword, with their effects depending on the method of administration and health status. The present findings provide a further ray of hope, but also a new challenge, for the use of KBs to prolong healthy life span.

## 2 METHODS

### 2.1 Ethics

The experimental protocols were approved by the Gene Recombination Experiment Safety Committee and Research Center for Animal Life Science of Shiga University of Medical Science.

### 2.2 Animal studies

Details on the creation of each transgenic mouse, the dietary intervention, and the measurement of each parameter are provided in the Supplemental Information.

### 2.3 Statistical analysis

Two‐way ANOVA followed by Tukey's *post hoc* test was used to determine the effects of genotype and treatment among multiple groups, and the unpaired Student's *t* test was used to compare two groups. Survival rates were determined using the Kaplan–Meier method and compared among the groups using the log‐rank test, with the Bonferroni correction. R statistical software version 1.55 (Vienna, Austria) was used for the analysis. *p* < 0.05 was considered to represent statistical significance. Each experiment was conducted twice, and the combined data were statistically analyzed.

## AUTHOR CONTRIBUTIONS

I.T., S.K., and H.M. designed the study. I.T. and H.T. performed the experiments. I.T., S.K., M.Y.‐Y., K.Y., S.K., Y.T.‐S., M.C.‐K., Y.F., E.N., and H.K. discussed and analyzed the data. I.T. and S.K. drafted the manuscript. All the authors revised the manuscript and approved the final version.

## CONFLICT OF INTEREST STATEMENT

The authors declare no competing interests.

## Supporting information


Appendix S1

## Data Availability

The datasets are available from the corresponding author on reasonable request.
