# Supplementary material for: Ketone bodies: A double‐edged sword for mammalian life span
Source: Aging Cell. 2023 Apr 14;22(6):e13833. doi: 10.1111/acel.13833 (PMC10265173; doi:10.1111/acel.13833)
Supplement: Supplementary file 1 — Appendix S1 [file ACEL-22-e13833-s001.pptx]

## Slide 1
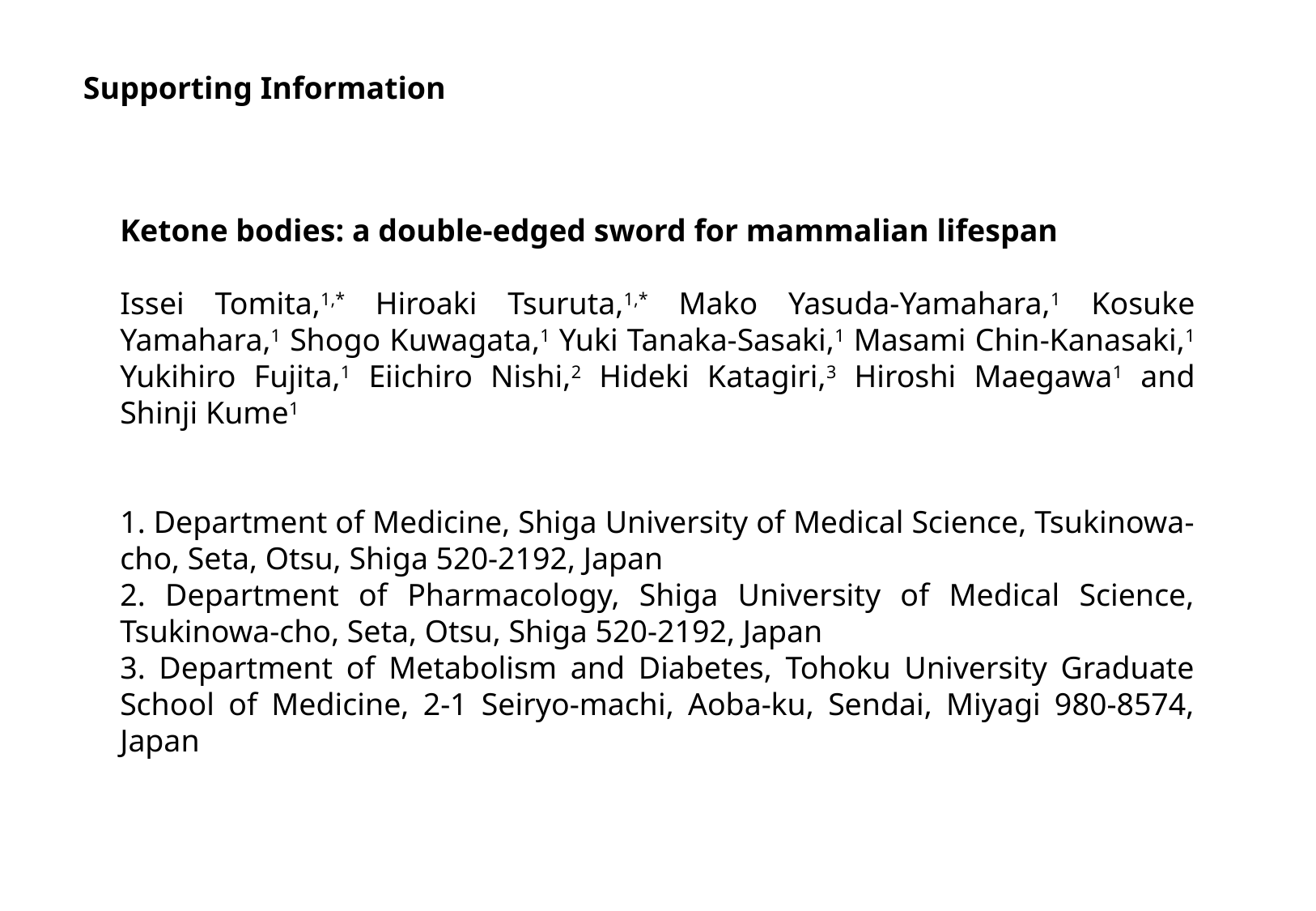

Supporting Information
Ketone bodies: a double-edged sword for mammalian lifespan
Issei Tomita,1,* Hiroaki Tsuruta,1,* Mako Yasuda-Yamahara,1 Kosuke Yamahara,1 Shogo Kuwagata,1 Yuki Tanaka-Sasaki,1 Masami Chin-Kanasaki,1 Yukihiro Fujita,1 Eiichiro Nishi,2 Hideki Katagiri,3 Hiroshi Maegawa1 and Shinji Kume1
1. Department of Medicine, Shiga University of Medical Science, Tsukinowa-cho, Seta, Otsu, Shiga 520-2192, Japan
2. Department of Pharmacology, Shiga University of Medical Science, Tsukinowa-cho, Seta, Otsu, Shiga 520-2192, Japan
3. Department of Metabolism and Diabetes, Tohoku University Graduate School of Medicine, 2-1 Seiryo-machi, Aoba-ku, Sendai, Miyagi 980-8574, Japan

## Slide 2
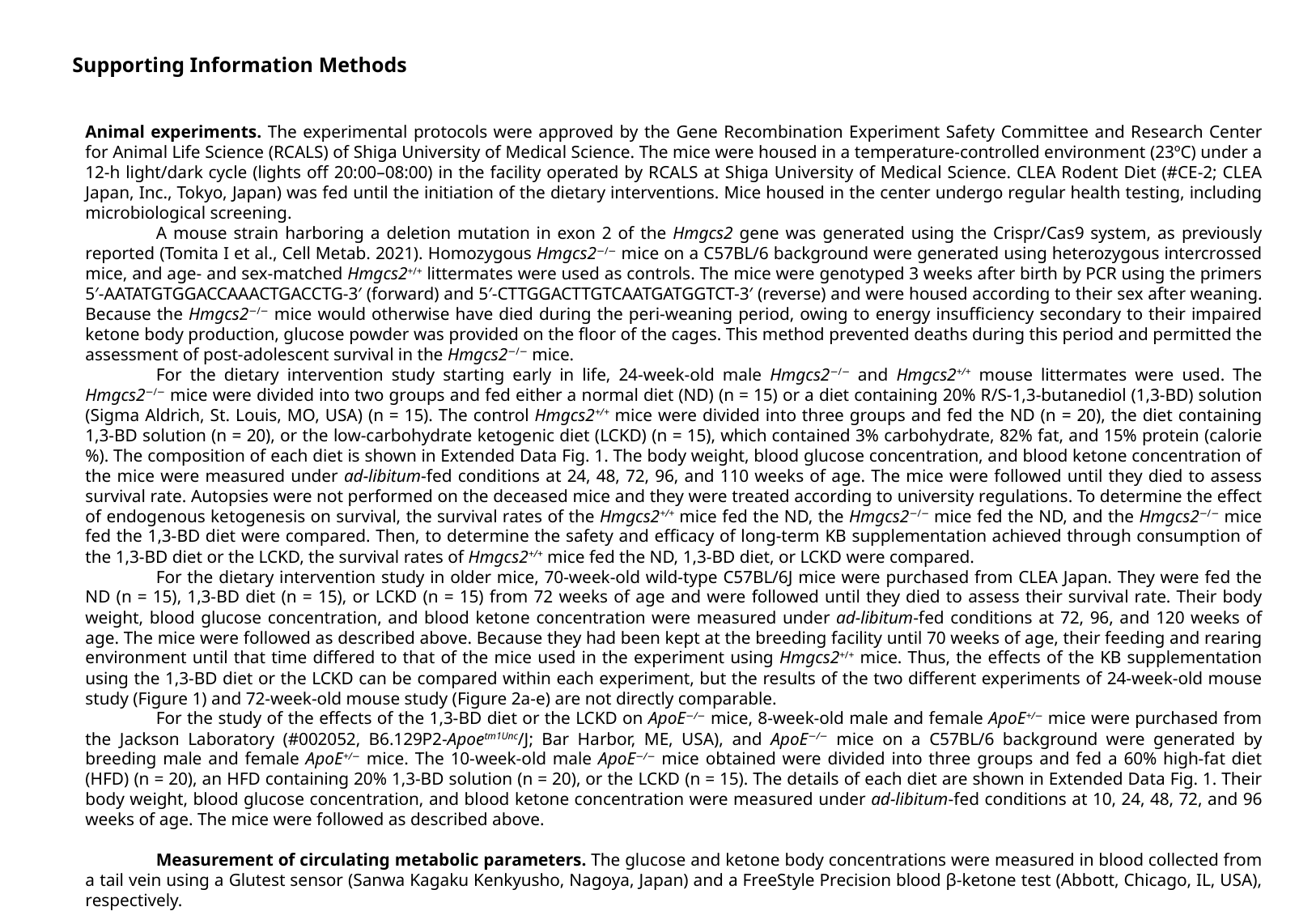

Supporting Information Methods
Animal experiments. The experimental protocols were approved by the Gene Recombination Experiment Safety Committee and Research Center for Animal Life Science (RCALS) of Shiga University of Medical Science. The mice were housed in a temperature-controlled environment (23ºC) under a 12-h light/dark cycle (lights off 20:00–08:00) in the facility operated by RCALS at Shiga University of Medical Science. CLEA Rodent Diet (#CE-2; CLEA Japan, Inc., Tokyo, Japan) was fed until the initiation of the dietary interventions. Mice housed in the center undergo regular health testing, including microbiological screening.
A mouse strain harboring a deletion mutation in exon 2 of the Hmgcs2 gene was generated using the Crispr/Cas9 system, as previously reported (Tomita I et al., Cell Metab. 2021). Homozygous Hmgcs2−/− mice on a C57BL/6 background were generated using heterozygous intercrossed mice, and age- and sex-matched Hmgcs2+/+ littermates were used as controls. The mice were genotyped 3 weeks after birth by PCR using the primers 5′-AATATGTGGACCAAACTGACCTG-3′ (forward) and 5′-CTTGGACTTGTCAATGATGGTCT-3′ (reverse) and were housed according to their sex after weaning. Because the Hmgcs2−/− mice would otherwise have died during the peri-weaning period, owing to energy insufficiency secondary to their impaired ketone body production, glucose powder was provided on the floor of the cages. This method prevented deaths during this period and permitted the assessment of post-adolescent survival in the Hmgcs2−/− mice.
For the dietary intervention study starting early in life, 24-week-old male Hmgcs2−/− and Hmgcs2+/+ mouse littermates were used. The Hmgcs2−/− mice were divided into two groups and fed either a normal diet (ND) (n = 15) or a diet containing 20% R/S-1,3-butanediol (1,3-BD) solution (Sigma Aldrich, St. Louis, MO, USA) (n = 15). The control Hmgcs2+/+ mice were divided into three groups and fed the ND (n = 20), the diet containing 1,3-BD solution (n = 20), or the low-carbohydrate ketogenic diet (LCKD) (n = 15), which contained 3% carbohydrate, 82% fat, and 15% protein (calorie %). The composition of each diet is shown in Extended Data Fig. 1. The body weight, blood glucose concentration, and blood ketone concentration of the mice were measured under ad-libitum-fed conditions at 24, 48, 72, 96, and 110 weeks of age. The mice were followed until they died to assess survival rate. Autopsies were not performed on the deceased mice and they were treated according to university regulations. To determine the effect of endogenous ketogenesis on survival, the survival rates of the Hmgcs2+/+ mice fed the ND, the Hmgcs2−/− mice fed the ND, and the Hmgcs2−/− mice fed the 1,3-BD diet were compared. Then, to determine the safety and efficacy of long-term KB supplementation achieved through consumption of the 1,3-BD diet or the LCKD, the survival rates of Hmgcs2+/+ mice fed the ND, 1,3-BD diet, or LCKD were compared.
For the dietary intervention study in older mice, 70-week-old wild-type C57BL/6J mice were purchased from CLEA Japan. They were fed the ND (n = 15), 1,3-BD diet (n = 15), or LCKD (n = 15) from 72 weeks of age and were followed until they died to assess their survival rate. Their body weight, blood glucose concentration, and blood ketone concentration were measured under ad-libitum-fed conditions at 72, 96, and 120 weeks of age. The mice were followed as described above. Because they had been kept at the breeding facility until 70 weeks of age, their feeding and rearing environment until that time differed to that of the mice used in the experiment using Hmgcs2+/+ mice. Thus, the effects of the KB supplementation using the 1,3-BD diet or the LCKD can be compared within each experiment, but the results of the two different experiments of 24-week-old mouse study (Figure 1) and 72-week-old mouse study (Figure 2a-e) are not directly comparable.
For the study of the effects of the 1,3-BD diet or the LCKD on ApoE−/− mice, 8-week-old male and female ApoE+/− mice were purchased from the Jackson Laboratory (#002052, B6.129P2-Apoetm1Unc/J; Bar Harbor, ME, USA), and ApoE−/− mice on a C57BL/6 background were generated by breeding male and female ApoE+/− mice. The 10-week-old male ApoE−/− mice obtained were divided into three groups and fed a 60% high-fat diet (HFD) (n = 20), an HFD containing 20% 1,3-BD solution (n = 20), or the LCKD (n = 15). The details of each diet are shown in Extended Data Fig. 1. Their body weight, blood glucose concentration, and blood ketone concentration were measured under ad-libitum-fed conditions at 10, 24, 48, 72, and 96 weeks of age. The mice were followed as described above.
Measurement of circulating metabolic parameters. The glucose and ketone body concentrations were measured in blood collected from a tail vein using a Glutest sensor (Sanwa Kagaku Kenkyusho, Nagoya, Japan) and a FreeStyle Precision blood β-ketone test (Abbott, Chicago, IL, USA), respectively.

## Slide 3
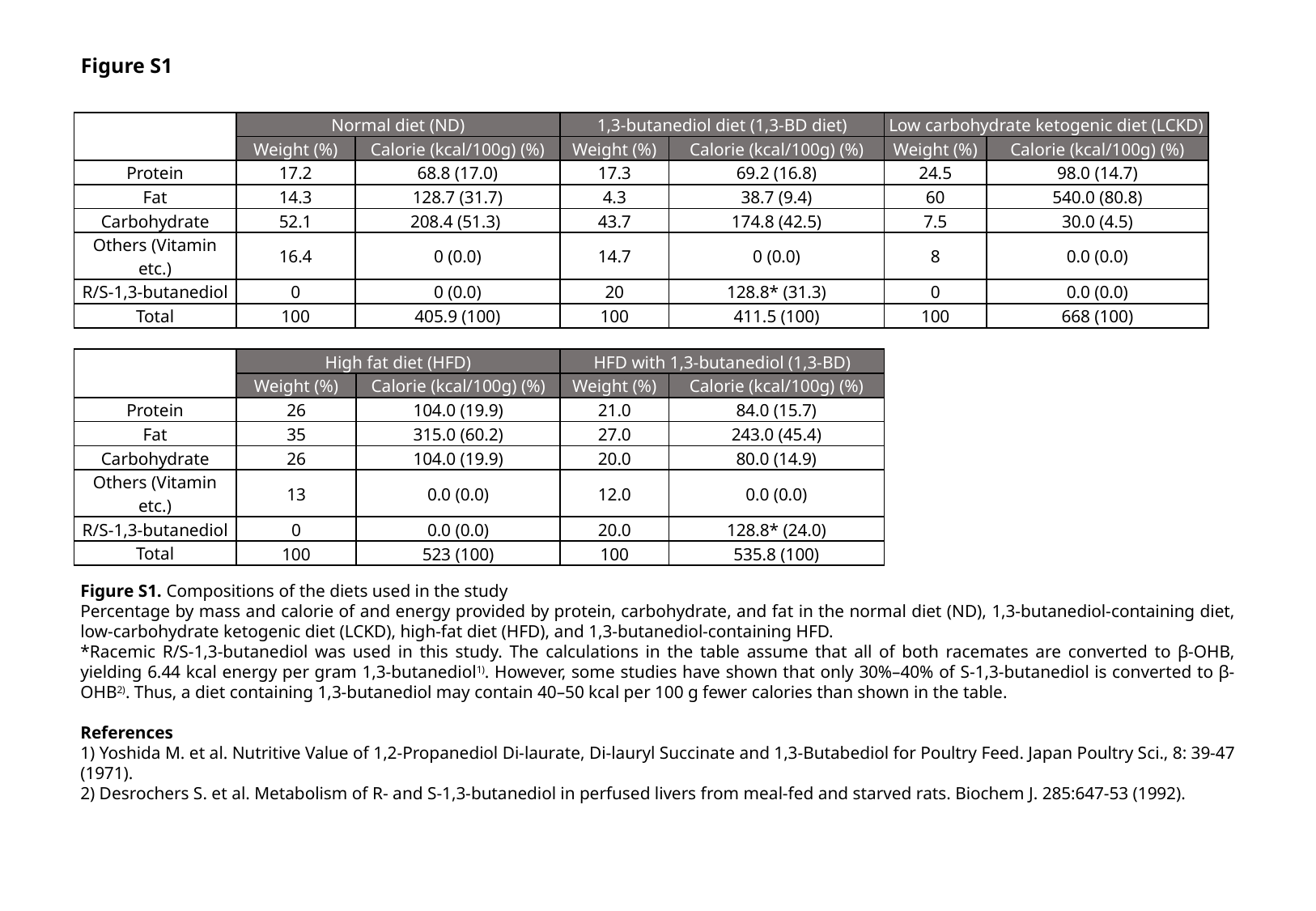

Figure S1
| | Normal diet (ND) | | 1,3-butanediol diet (1,3-BD diet) | | Low carbohydrate ketogenic diet (LCKD) | |
| --- | --- | --- | --- | --- | --- | --- |
| | Weight (%) | Calorie (kcal/100g) (%) | Weight (%) | Calorie (kcal/100g) (%) | Weight (%) | Calorie (kcal/100g) (%) |
| Protein | 17.2 | 68.8 (17.0) | 17.3 | 69.2 (16.8) | 24.5 | 98.0 (14.7) |
| Fat | 14.3 | 128.7 (31.7) | 4.3 | 38.7 (9.4) | 60 | 540.0 (80.8) |
| Carbohydrate | 52.1 | 208.4 (51.3) | 43.7 | 174.8 (42.5) | 7.5 | 30.0 (4.5) |
| Others (Vitamin etc.) | 16.4 | 0 (0.0) | 14.7 | 0 (0.0) | 8 | 0.0 (0.0) |
| R/S-1,3-butanediol | 0 | 0 (0.0) | 20 | 128.8\* (31.3) | 0 | 0.0 (0.0) |
| Total | 100 | 405.9 (100) | 100 | 411.5 (100) | 100 | 668 (100) |
| | High fat diet (HFD) | | HFD with 1,3-butanediol (1,3-BD) | |
| --- | --- | --- | --- | --- |
| | Weight (%) | Calorie (kcal/100g) (%) | Weight (%) | Calorie (kcal/100g) (%) |
| Protein | 26 | 104.0 (19.9) | 21.0 | 84.0 (15.7) |
| Fat | 35 | 315.0 (60.2) | 27.0 | 243.0 (45.4) |
| Carbohydrate | 26 | 104.0 (19.9) | 20.0 | 80.0 (14.9) |
| Others (Vitamin etc.) | 13 | 0.0 (0.0) | 12.0 | 0.0 (0.0) |
| R/S-1,3-butanediol | 0 | 0.0 (0.0) | 20.0 | 128.8\* (24.0) |
| Total | 100 | 523 (100) | 100 | 535.8 (100) |
Figure S1. Compositions of the diets used in the study
Percentage by mass and calorie of and energy provided by protein, carbohydrate, and fat in the normal diet (ND), 1,3-butanediol-containing diet, low-carbohydrate ketogenic diet (LCKD), high-fat diet (HFD), and 1,3-butanediol-containing HFD.
*Racemic R/S-1,3-butanediol was used in this study. The calculations in the table assume that all of both racemates are converted to β-OHB, yielding 6.44 kcal energy per gram 1,3-butanediol1). However, some studies have shown that only 30%–40% of S-1,3-butanediol is converted to β-OHB2). Thus, a diet containing 1,3-butanediol may contain 40–50 kcal per 100 g fewer calories than shown in the table.
References
1) Yoshida M. et al. Nutritive Value of 1,2-Propanediol Di-laurate, Di-lauryl Succinate and 1,3-Butabediol for Poultry Feed. Japan Poultry Sci., 8: 39-47 (1971).
2) Desrochers S. et al. Metabolism of R- and S-1,3-butanediol in perfused livers from meal-fed and starved rats. Biochem J. 285:647-53 (1992).

## Slide 4
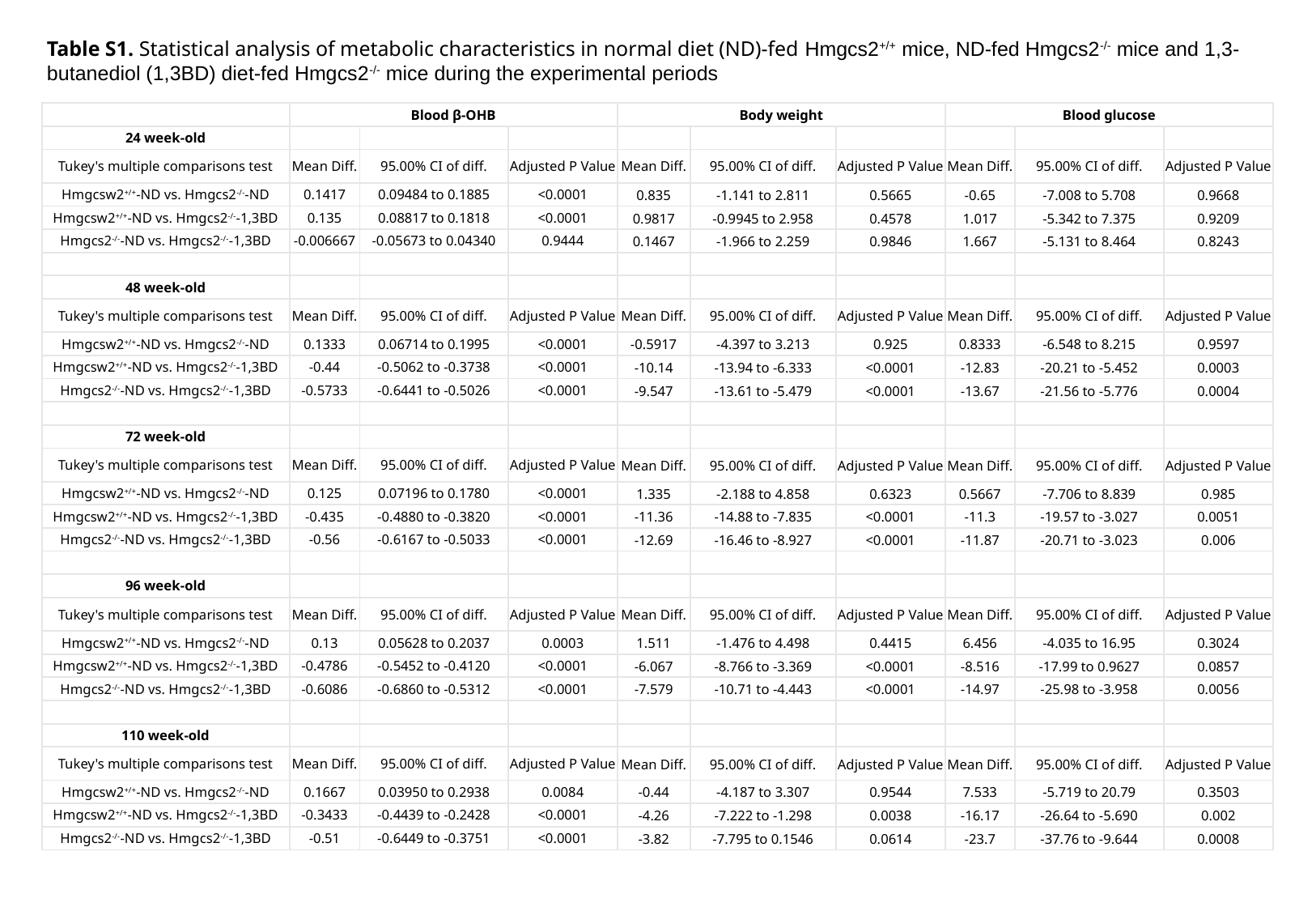

Table S1. Statistical analysis of metabolic characteristics in normal diet (ND)-fed Hmgcs2+/+ mice, ND-fed Hmgcs2-/- mice and 1,3-butanediol (1,3BD) diet-fed Hmgcs2-/- mice during the experimental periods
| | Blood β-OHB | | | Body weight | | | Blood glucose | | |
| --- | --- | --- | --- | --- | --- | --- | --- | --- | --- |
| 24 week-old | | | | | | | | | |
| Tukey's multiple comparisons test | Mean Diff. | 95.00% CI of diff. | Adjusted P Value | Mean Diff. | 95.00% CI of diff. | Adjusted P Value | Mean Diff. | 95.00% CI of diff. | Adjusted P Value |
| Hmgcsw2+/+-ND vs. Hmgcs2-/--ND | 0.1417 | 0.09484 to 0.1885 | <0.0001 | 0.835 | -1.141 to 2.811 | 0.5665 | -0.65 | -7.008 to 5.708 | 0.9668 |
| Hmgcsw2+/+-ND vs. Hmgcs2-/--1,3BD | 0.135 | 0.08817 to 0.1818 | <0.0001 | 0.9817 | -0.9945 to 2.958 | 0.4578 | 1.017 | -5.342 to 7.375 | 0.9209 |
| Hmgcs2-/--ND vs. Hmgcs2-/--1,3BD | -0.006667 | -0.05673 to 0.04340 | 0.9444 | 0.1467 | -1.966 to 2.259 | 0.9846 | 1.667 | -5.131 to 8.464 | 0.8243 |
| | | | | | | | | | |
| 48 week-old | | | | | | | | | |
| Tukey's multiple comparisons test | Mean Diff. | 95.00% CI of diff. | Adjusted P Value | Mean Diff. | 95.00% CI of diff. | Adjusted P Value | Mean Diff. | 95.00% CI of diff. | Adjusted P Value |
| Hmgcsw2+/+-ND vs. Hmgcs2-/--ND | 0.1333 | 0.06714 to 0.1995 | <0.0001 | -0.5917 | -4.397 to 3.213 | 0.925 | 0.8333 | -6.548 to 8.215 | 0.9597 |
| Hmgcsw2+/+-ND vs. Hmgcs2-/--1,3BD | -0.44 | -0.5062 to -0.3738 | <0.0001 | -10.14 | -13.94 to -6.333 | <0.0001 | -12.83 | -20.21 to -5.452 | 0.0003 |
| Hmgcs2-/--ND vs. Hmgcs2-/--1,3BD | -0.5733 | -0.6441 to -0.5026 | <0.0001 | -9.547 | -13.61 to -5.479 | <0.0001 | -13.67 | -21.56 to -5.776 | 0.0004 |
| | | | | | | | | | |
| 72 week-old | | | | | | | | | |
| Tukey's multiple comparisons test | Mean Diff. | 95.00% CI of diff. | Adjusted P Value | Mean Diff. | 95.00% CI of diff. | Adjusted P Value | Mean Diff. | 95.00% CI of diff. | Adjusted P Value |
| Hmgcsw2+/+-ND vs. Hmgcs2-/--ND | 0.125 | 0.07196 to 0.1780 | <0.0001 | 1.335 | -2.188 to 4.858 | 0.6323 | 0.5667 | -7.706 to 8.839 | 0.985 |
| Hmgcsw2+/+-ND vs. Hmgcs2-/--1,3BD | -0.435 | -0.4880 to -0.3820 | <0.0001 | -11.36 | -14.88 to -7.835 | <0.0001 | -11.3 | -19.57 to -3.027 | 0.0051 |
| Hmgcs2-/--ND vs. Hmgcs2-/--1,3BD | -0.56 | -0.6167 to -0.5033 | <0.0001 | -12.69 | -16.46 to -8.927 | <0.0001 | -11.87 | -20.71 to -3.023 | 0.006 |
| | | | | | | | | | |
| 96 week-old | | | | | | | | | |
| Tukey's multiple comparisons test | Mean Diff. | 95.00% CI of diff. | Adjusted P Value | Mean Diff. | 95.00% CI of diff. | Adjusted P Value | Mean Diff. | 95.00% CI of diff. | Adjusted P Value |
| Hmgcsw2+/+-ND vs. Hmgcs2-/--ND | 0.13 | 0.05628 to 0.2037 | 0.0003 | 1.511 | -1.476 to 4.498 | 0.4415 | 6.456 | -4.035 to 16.95 | 0.3024 |
| Hmgcsw2+/+-ND vs. Hmgcs2-/--1,3BD | -0.4786 | -0.5452 to -0.4120 | <0.0001 | -6.067 | -8.766 to -3.369 | <0.0001 | -8.516 | -17.99 to 0.9627 | 0.0857 |
| Hmgcs2-/--ND vs. Hmgcs2-/--1,3BD | -0.6086 | -0.6860 to -0.5312 | <0.0001 | -7.579 | -10.71 to -4.443 | <0.0001 | -14.97 | -25.98 to -3.958 | 0.0056 |
| | | | | | | | | | |
| 110 week-old | | | | | | | | | |
| Tukey's multiple comparisons test | Mean Diff. | 95.00% CI of diff. | Adjusted P Value | Mean Diff. | 95.00% CI of diff. | Adjusted P Value | Mean Diff. | 95.00% CI of diff. | Adjusted P Value |
| Hmgcsw2+/+-ND vs. Hmgcs2-/--ND | 0.1667 | 0.03950 to 0.2938 | 0.0084 | -0.44 | -4.187 to 3.307 | 0.9544 | 7.533 | -5.719 to 20.79 | 0.3503 |
| Hmgcsw2+/+-ND vs. Hmgcs2-/--1,3BD | -0.3433 | -0.4439 to -0.2428 | <0.0001 | -4.26 | -7.222 to -1.298 | 0.0038 | -16.17 | -26.64 to -5.690 | 0.002 |
| Hmgcs2-/--ND vs. Hmgcs2-/--1,3BD | -0.51 | -0.6449 to -0.3751 | <0.0001 | -3.82 | -7.795 to 0.1546 | 0.0614 | -23.7 | -37.76 to -9.644 | 0.0008 |

## Slide 5
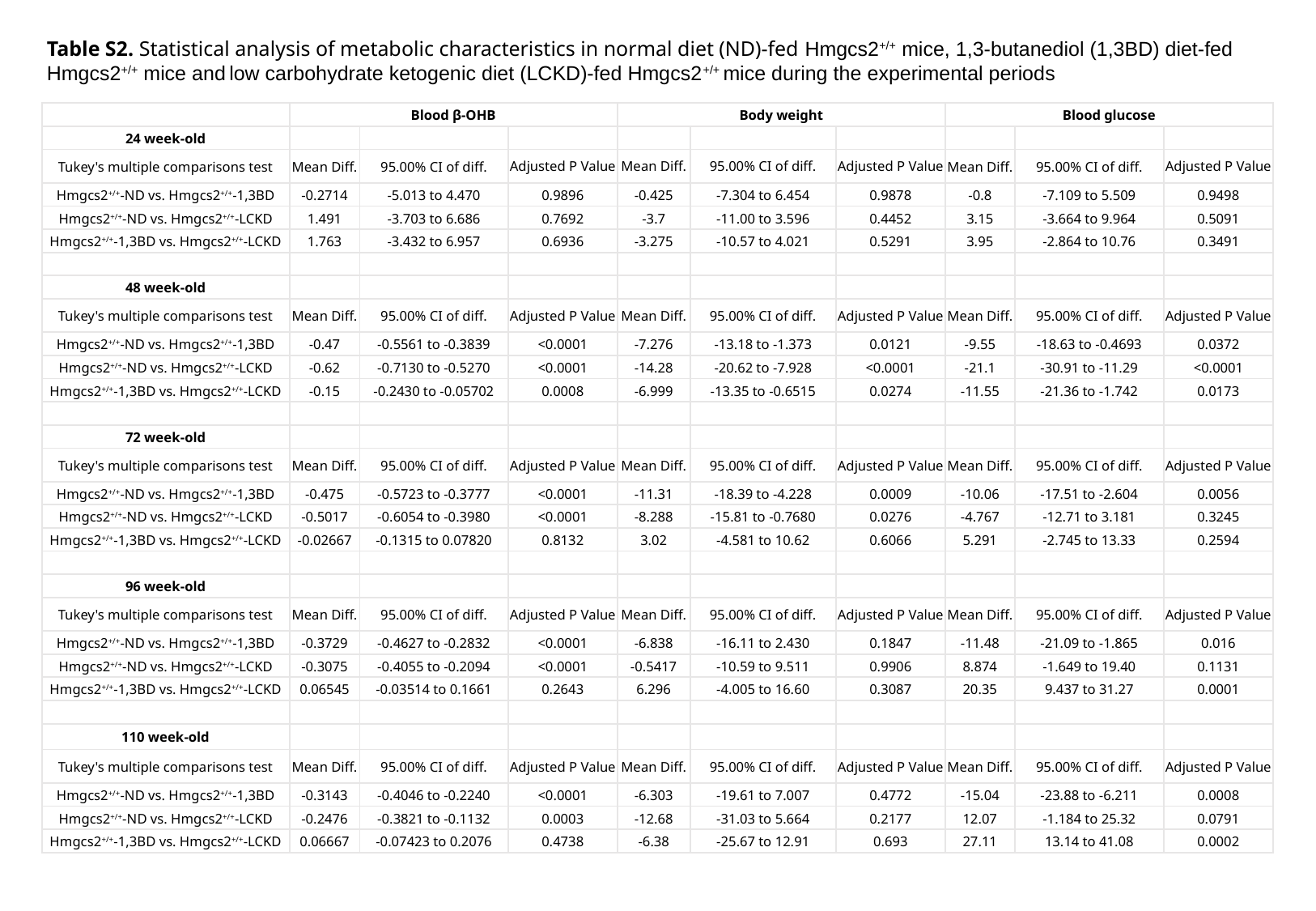

Table S2. Statistical analysis of metabolic characteristics in normal diet (ND)-fed Hmgcs2+/+ mice, 1,3-butanediol (1,3BD) diet-fed Hmgcs2+/+ mice and low carbohydrate ketogenic diet (LCKD)-fed Hmgcs2+/+ mice during the experimental periods
| | Blood β-OHB | | | Body weight | | | Blood glucose | | |
| --- | --- | --- | --- | --- | --- | --- | --- | --- | --- |
| 24 week-old | | | | | | | | | |
| Tukey's multiple comparisons test | Mean Diff. | 95.00% CI of diff. | Adjusted P Value | Mean Diff. | 95.00% CI of diff. | Adjusted P Value | Mean Diff. | 95.00% CI of diff. | Adjusted P Value |
| Hmgcs2+/+-ND vs. Hmgcs2+/+-1,3BD | -0.2714 | -5.013 to 4.470 | 0.9896 | -0.425 | -7.304 to 6.454 | 0.9878 | -0.8 | -7.109 to 5.509 | 0.9498 |
| Hmgcs2+/+-ND vs. Hmgcs2+/+-LCKD | 1.491 | -3.703 to 6.686 | 0.7692 | -3.7 | -11.00 to 3.596 | 0.4452 | 3.15 | -3.664 to 9.964 | 0.5091 |
| Hmgcs2+/+-1,3BD vs. Hmgcs2+/+-LCKD | 1.763 | -3.432 to 6.957 | 0.6936 | -3.275 | -10.57 to 4.021 | 0.5291 | 3.95 | -2.864 to 10.76 | 0.3491 |
| | | | | | | | | | |
| 48 week-old | | | | | | | | | |
| Tukey's multiple comparisons test | Mean Diff. | 95.00% CI of diff. | Adjusted P Value | Mean Diff. | 95.00% CI of diff. | Adjusted P Value | Mean Diff. | 95.00% CI of diff. | Adjusted P Value |
| Hmgcs2+/+-ND vs. Hmgcs2+/+-1,3BD | -0.47 | -0.5561 to -0.3839 | <0.0001 | -7.276 | -13.18 to -1.373 | 0.0121 | -9.55 | -18.63 to -0.4693 | 0.0372 |
| Hmgcs2+/+-ND vs. Hmgcs2+/+-LCKD | -0.62 | -0.7130 to -0.5270 | <0.0001 | -14.28 | -20.62 to -7.928 | <0.0001 | -21.1 | -30.91 to -11.29 | <0.0001 |
| Hmgcs2+/+-1,3BD vs. Hmgcs2+/+-LCKD | -0.15 | -0.2430 to -0.05702 | 0.0008 | -6.999 | -13.35 to -0.6515 | 0.0274 | -11.55 | -21.36 to -1.742 | 0.0173 |
| | | | | | | | | | |
| 72 week-old | | | | | | | | | |
| Tukey's multiple comparisons test | Mean Diff. | 95.00% CI of diff. | Adjusted P Value | Mean Diff. | 95.00% CI of diff. | Adjusted P Value | Mean Diff. | 95.00% CI of diff. | Adjusted P Value |
| Hmgcs2+/+-ND vs. Hmgcs2+/+-1,3BD | -0.475 | -0.5723 to -0.3777 | <0.0001 | -11.31 | -18.39 to -4.228 | 0.0009 | -10.06 | -17.51 to -2.604 | 0.0056 |
| Hmgcs2+/+-ND vs. Hmgcs2+/+-LCKD | -0.5017 | -0.6054 to -0.3980 | <0.0001 | -8.288 | -15.81 to -0.7680 | 0.0276 | -4.767 | -12.71 to 3.181 | 0.3245 |
| Hmgcs2+/+-1,3BD vs. Hmgcs2+/+-LCKD | -0.02667 | -0.1315 to 0.07820 | 0.8132 | 3.02 | -4.581 to 10.62 | 0.6066 | 5.291 | -2.745 to 13.33 | 0.2594 |
| | | | | | | | | | |
| 96 week-old | | | | | | | | | |
| Tukey's multiple comparisons test | Mean Diff. | 95.00% CI of diff. | Adjusted P Value | Mean Diff. | 95.00% CI of diff. | Adjusted P Value | Mean Diff. | 95.00% CI of diff. | Adjusted P Value |
| Hmgcs2+/+-ND vs. Hmgcs2+/+-1,3BD | -0.3729 | -0.4627 to -0.2832 | <0.0001 | -6.838 | -16.11 to 2.430 | 0.1847 | -11.48 | -21.09 to -1.865 | 0.016 |
| Hmgcs2+/+-ND vs. Hmgcs2+/+-LCKD | -0.3075 | -0.4055 to -0.2094 | <0.0001 | -0.5417 | -10.59 to 9.511 | 0.9906 | 8.874 | -1.649 to 19.40 | 0.1131 |
| Hmgcs2+/+-1,3BD vs. Hmgcs2+/+-LCKD | 0.06545 | -0.03514 to 0.1661 | 0.2643 | 6.296 | -4.005 to 16.60 | 0.3087 | 20.35 | 9.437 to 31.27 | 0.0001 |
| | | | | | | | | | |
| 110 week-old | | | | | | | | | |
| Tukey's multiple comparisons test | Mean Diff. | 95.00% CI of diff. | Adjusted P Value | Mean Diff. | 95.00% CI of diff. | Adjusted P Value | Mean Diff. | 95.00% CI of diff. | Adjusted P Value |
| Hmgcs2+/+-ND vs. Hmgcs2+/+-1,3BD | -0.3143 | -0.4046 to -0.2240 | <0.0001 | -6.303 | -19.61 to 7.007 | 0.4772 | -15.04 | -23.88 to -6.211 | 0.0008 |
| Hmgcs2+/+-ND vs. Hmgcs2+/+-LCKD | -0.2476 | -0.3821 to -0.1132 | 0.0003 | -12.68 | -31.03 to 5.664 | 0.2177 | 12.07 | -1.184 to 25.32 | 0.0791 |
| Hmgcs2+/+-1,3BD vs. Hmgcs2+/+-LCKD | 0.06667 | -0.07423 to 0.2076 | 0.4738 | -6.38 | -25.67 to 12.91 | 0.693 | 27.11 | 13.14 to 41.08 | 0.0002 |

## Slide 6
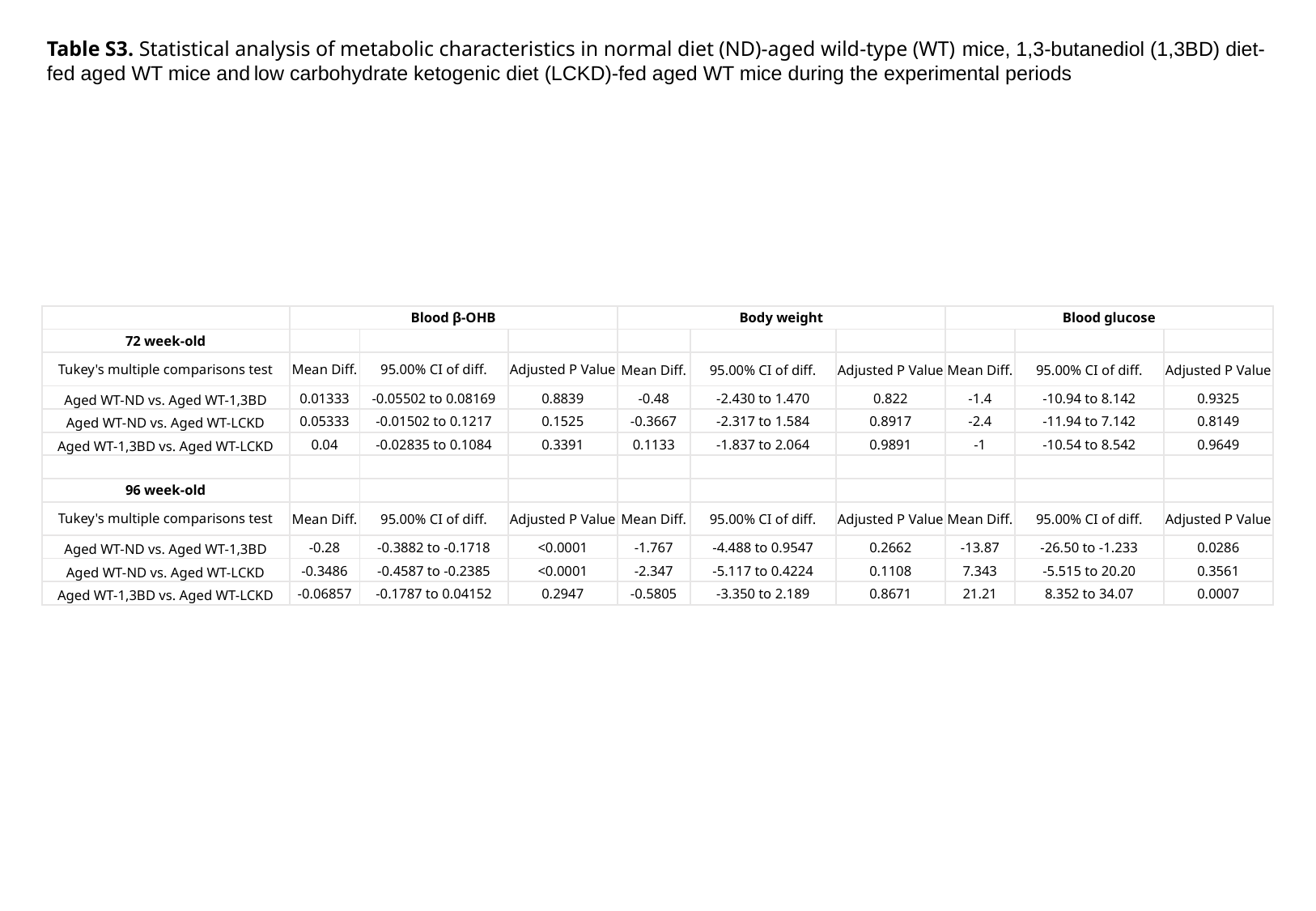

Table S3. Statistical analysis of metabolic characteristics in normal diet (ND)-aged wild-type (WT) mice, 1,3-butanediol (1,3BD) diet-fed aged WT mice and low carbohydrate ketogenic diet (LCKD)-fed aged WT mice during the experimental periods
| | Blood β-OHB | | | Body weight | | | Blood glucose | | |
| --- | --- | --- | --- | --- | --- | --- | --- | --- | --- |
| 72 week-old | | | | | | | | | |
| Tukey's multiple comparisons test | Mean Diff. | 95.00% CI of diff. | Adjusted P Value | Mean Diff. | 95.00% CI of diff. | Adjusted P Value | Mean Diff. | 95.00% CI of diff. | Adjusted P Value |
| Aged WT-ND vs. Aged WT-1,3BD | 0.01333 | -0.05502 to 0.08169 | 0.8839 | -0.48 | -2.430 to 1.470 | 0.822 | -1.4 | -10.94 to 8.142 | 0.9325 |
| Aged WT-ND vs. Aged WT-LCKD | 0.05333 | -0.01502 to 0.1217 | 0.1525 | -0.3667 | -2.317 to 1.584 | 0.8917 | -2.4 | -11.94 to 7.142 | 0.8149 |
| Aged WT-1,3BD vs. Aged WT-LCKD | 0.04 | -0.02835 to 0.1084 | 0.3391 | 0.1133 | -1.837 to 2.064 | 0.9891 | -1 | -10.54 to 8.542 | 0.9649 |
| | | | | | | | | | |
| 96 week-old | | | | | | | | | |
| Tukey's multiple comparisons test | Mean Diff. | 95.00% CI of diff. | Adjusted P Value | Mean Diff. | 95.00% CI of diff. | Adjusted P Value | Mean Diff. | 95.00% CI of diff. | Adjusted P Value |
| Aged WT-ND vs. Aged WT-1,3BD | -0.28 | -0.3882 to -0.1718 | <0.0001 | -1.767 | -4.488 to 0.9547 | 0.2662 | -13.87 | -26.50 to -1.233 | 0.0286 |
| Aged WT-ND vs. Aged WT-LCKD | -0.3486 | -0.4587 to -0.2385 | <0.0001 | -2.347 | -5.117 to 0.4224 | 0.1108 | 7.343 | -5.515 to 20.20 | 0.3561 |
| Aged WT-1,3BD vs. Aged WT-LCKD | -0.06857 | -0.1787 to 0.04152 | 0.2947 | -0.5805 | -3.350 to 2.189 | 0.8671 | 21.21 | 8.352 to 34.07 | 0.0007 |

## Slide 7
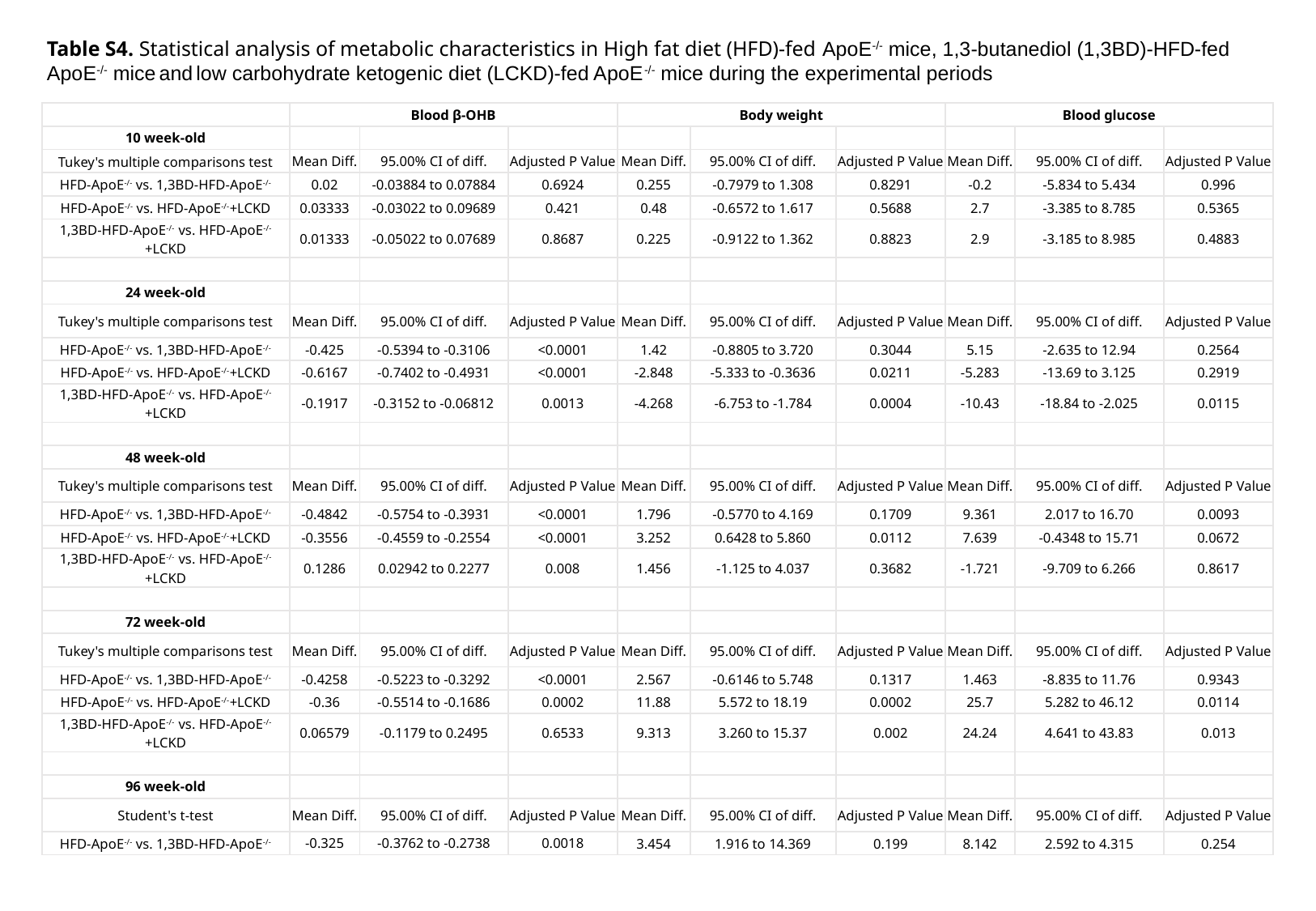

Table S4. Statistical analysis of metabolic characteristics in High fat diet (HFD)-fed ApoE-/- mice, 1,3-butanediol (1,3BD)-HFD-fed ApoE-/- mice and low carbohydrate ketogenic diet (LCKD)-fed ApoE-/- mice during the experimental periods
| | Blood β-OHB | | | Body weight | | | Blood glucose | | |
| --- | --- | --- | --- | --- | --- | --- | --- | --- | --- |
| 10 week-old | | | | | | | | | |
| Tukey's multiple comparisons test | Mean Diff. | 95.00% CI of diff. | Adjusted P Value | Mean Diff. | 95.00% CI of diff. | Adjusted P Value | Mean Diff. | 95.00% CI of diff. | Adjusted P Value |
| HFD-ApoE-/- vs. 1,3BD-HFD-ApoE-/- | 0.02 | -0.03884 to 0.07884 | 0.6924 | 0.255 | -0.7979 to 1.308 | 0.8291 | -0.2 | -5.834 to 5.434 | 0.996 |
| HFD-ApoE-/- vs. HFD-ApoE-/-+LCKD | 0.03333 | -0.03022 to 0.09689 | 0.421 | 0.48 | -0.6572 to 1.617 | 0.5688 | 2.7 | -3.385 to 8.785 | 0.5365 |
| 1,3BD-HFD-ApoE-/- vs. HFD-ApoE-/-+LCKD | 0.01333 | -0.05022 to 0.07689 | 0.8687 | 0.225 | -0.9122 to 1.362 | 0.8823 | 2.9 | -3.185 to 8.985 | 0.4883 |
| | | | | | | | | | |
| 24 week-old | | | | | | | | | |
| Tukey's multiple comparisons test | Mean Diff. | 95.00% CI of diff. | Adjusted P Value | Mean Diff. | 95.00% CI of diff. | Adjusted P Value | Mean Diff. | 95.00% CI of diff. | Adjusted P Value |
| HFD-ApoE-/- vs. 1,3BD-HFD-ApoE-/- | -0.425 | -0.5394 to -0.3106 | <0.0001 | 1.42 | -0.8805 to 3.720 | 0.3044 | 5.15 | -2.635 to 12.94 | 0.2564 |
| HFD-ApoE-/- vs. HFD-ApoE-/-+LCKD | -0.6167 | -0.7402 to -0.4931 | <0.0001 | -2.848 | -5.333 to -0.3636 | 0.0211 | -5.283 | -13.69 to 3.125 | 0.2919 |
| 1,3BD-HFD-ApoE-/- vs. HFD-ApoE-/-+LCKD | -0.1917 | -0.3152 to -0.06812 | 0.0013 | -4.268 | -6.753 to -1.784 | 0.0004 | -10.43 | -18.84 to -2.025 | 0.0115 |
| | | | | | | | | | |
| 48 week-old | | | | | | | | | |
| Tukey's multiple comparisons test | Mean Diff. | 95.00% CI of diff. | Adjusted P Value | Mean Diff. | 95.00% CI of diff. | Adjusted P Value | Mean Diff. | 95.00% CI of diff. | Adjusted P Value |
| HFD-ApoE-/- vs. 1,3BD-HFD-ApoE-/- | -0.4842 | -0.5754 to -0.3931 | <0.0001 | 1.796 | -0.5770 to 4.169 | 0.1709 | 9.361 | 2.017 to 16.70 | 0.0093 |
| HFD-ApoE-/- vs. HFD-ApoE-/-+LCKD | -0.3556 | -0.4559 to -0.2554 | <0.0001 | 3.252 | 0.6428 to 5.860 | 0.0112 | 7.639 | -0.4348 to 15.71 | 0.0672 |
| 1,3BD-HFD-ApoE-/- vs. HFD-ApoE-/-+LCKD | 0.1286 | 0.02942 to 0.2277 | 0.008 | 1.456 | -1.125 to 4.037 | 0.3682 | -1.721 | -9.709 to 6.266 | 0.8617 |
| | | | | | | | | | |
| 72 week-old | | | | | | | | | |
| Tukey's multiple comparisons test | Mean Diff. | 95.00% CI of diff. | Adjusted P Value | Mean Diff. | 95.00% CI of diff. | Adjusted P Value | Mean Diff. | 95.00% CI of diff. | Adjusted P Value |
| HFD-ApoE-/- vs. 1,3BD-HFD-ApoE-/- | -0.4258 | -0.5223 to -0.3292 | <0.0001 | 2.567 | -0.6146 to 5.748 | 0.1317 | 1.463 | -8.835 to 11.76 | 0.9343 |
| HFD-ApoE-/- vs. HFD-ApoE-/-+LCKD | -0.36 | -0.5514 to -0.1686 | 0.0002 | 11.88 | 5.572 to 18.19 | 0.0002 | 25.7 | 5.282 to 46.12 | 0.0114 |
| 1,3BD-HFD-ApoE-/- vs. HFD-ApoE-/-+LCKD | 0.06579 | -0.1179 to 0.2495 | 0.6533 | 9.313 | 3.260 to 15.37 | 0.002 | 24.24 | 4.641 to 43.83 | 0.013 |
| | | | | | | | | | |
| 96 week-old | | | | | | | | | |
| Student's t-test | Mean Diff. | 95.00% CI of diff. | Adjusted P Value | Mean Diff. | 95.00% CI of diff. | Adjusted P Value | Mean Diff. | 95.00% CI of diff. | Adjusted P Value |
| HFD-ApoE-/- vs. 1,3BD-HFD-ApoE-/- | -0.325 | -0.3762 to -0.2738 | 0.0018 | 3.454 | 1.916 to 14.369 | 0.199 | 8.142 | 2.592 to 4.315 | 0.254 |
